# Availability and utilization of oral healthcare services at rural community health centers in South India: a mixed methods study

**DOI:** 10.1186/s12903-025-06327-1

**Published:** 2025-07-01

**Authors:** Madhuri Panditi, Anusha K., Edukondal Palle, Prakash Babu Kodali

**Affiliations:** 1 Department of Public Health and Community Medicine, Central University of Kerala, Kasaragod, Kerala India; 2 Center for Tobacco Control Research and Education, University of California, San Francisco, USA

**Keywords:** Community health centers, Dentist, Healthcare access, Oral health

## Abstract

**Background:**

The National Oral Health Programme, launched by the Government of India, aims to provide comprehensive oral healthcare through public healthcare system. We conducted this study to assess availability and utilization of oral healthcare services at Community Health Centers (CHCs) in Prakasam district, Andhra Pradesh, India.

**Methods:**

Between January-June 2024, we conducted a mixed methods study comprising of facility survey of CHCs (*n* = 12), in-depth interview of CHC dentists (*n* = 12), and survey of individuals from randomly sampled households in the CHC catchment areas (*n* = 360). Facility survey and in-depth interviews were employed to study the availability of oral healthcare services, while household survey enabled assessment of their utilization. Survey data were analyzed employing descriptive and multivariate procedures. Qualitative interviews were analyzed using thematic analysis. We computed Adjusted odds ratios (AORs) for identifying factors associated with oral healthcare utilization.

**Results:**

All CHCs had a dentist, dental room, and dental chair, but none had dental assistants/hygienists. Services were limited to health education, scaling and root planing, and restorations. Demand deficit, health system preparedness, and operational constraints emerged as key themes. Only 13.9% sought oral healthcare from CHCs in the past year. Multiple symptoms (AOR = 3.19), awareness (AOR = 10.35), and perceived accessibility (AOR = 4.89) were significantly associated with oral healthcare utilization.

**Conclusions:**

Utilization of oral healthcare services is limited. Improving service utilization requires investment in infrastructure, human resources, and enhancing demand through education and outreach.

**Supplementary Information:**

The online version contains supplementary material available at 10.1186/s12903-025-06327-1.

## Background

Oral healthcare is less prioritized in low- and middle-income countries (LMICs), despite nearly 3.5 billion people being affected by oral diseases globally [1]. In India, common oral health issues include dental caries, toothache, gingival bleeding, loose teeth, and gingival sensitivity [2–4]. A high prevalence of tobacco use, dental fluorosis, and poor diets contribute to the epidemic proportions of oral health problems in India [2]. Oral healthcare in India is primarily provided by dentists with a Bachelor of Dental Surgery (BDS) qualification supported by Dental Assistant, Dental Hygienist, and Dental Technicians with a relevant diploma. There are 278,687 registered dentists across the country with a dentist to population ratio of 1:5000 [5]. Oral healthcare services are majorly accessed from private providers often resulting in catastrophic health expenditures for poor and vulnerable households [6]. 

In 2014-15, the Government of India launched National Oral Health Programme (NOHP) with an objective of integrating oral health promotion and preventive services into general healthcare by establishing dental units at public healthcare facilities [2]. India’s public healthcare system follows a three-tier hierarchy with Ayushman Arogya Mandir/ Primary Health Centers (PHCs) at the primary healthcare level, the Community Health Centers (CHCs) at the secondary healthcare level, and district hospitals/medical colleges/specialty hospitals at the tertiary healthcare level [5, 7–9]. The PHCs are expected to provide basic oral healthcare services, oral cancer screening, and organize school health education and dental outreach programmes [8]. The tertiary care centers like district hospitals are expected to undertake dental restoration, root canal treatments, maxilla facial and periodontal surgeries, apicectomy and gingivectomy etc [9]. While presence of a dentist at PHC is desired it is not essential [8], the CHCs are expected to have dedicated dental staff and serve as focal points to house dental units and provide comprehensive oral healthcare services including dental outpatient department (OPD) services (at least 20 per day), root canal treatments, routine and emergency extractions, fillings, and disimpactions, while also supporting dental outreach at PHCs [7]. The Indian Public Health Standards (IPHS) 2022 establish guidelines for equipping CHCs with essential human resources—such as a dentist and dental assistant—as well as critical infrastructure, including a dental chair, dental X-ray equipment, and materials like glass ionomer cement (GIC) [7]. However, the evidence on availability of oral healthcare services, their provision and utilization at CHC level is limited [10]. 

A 2022 meta-analysis reported a 23.96% pooled prevalence for the utilization of dental care among adults in India [11]. Previous research across Indian districts reported that nearly two-thirds had not visited a dentist in the last 12 months [12–15]. Healthcare utilization is influenced by need, individual predisposing factors, and enabling factors [16, 17]. In context of oral healthcare, these could be oral health symptoms (need), perceptions about oral health, and awareness of oral healthcare services (predisposing factors) and affordability and accessibility of oral healthcare services (enabling factors) [10–12, 17, 18]. Studies have also reported the role of socio-demographic factors (such as age, gender, income status of the households and education level of the individual) in accessing oral healthcare services [12, 13]. 

A decade since the implementation of the NOHP, there are limited comprehensive studies evaluating the availability and utilization of oral healthcare services at the CHCs that were upgraded with dental units under the programme. Our research examined (i) the availability of oral healthcare services and resources to support them (human resources and infrastructure), and (ii) factors influencing availability of oral healthcare services at CHCs. We also investigated the utilization of oral healthcare services provided by the CHCs and the factors (socio-demographic, need, predisposing and enabling) influencing their utilization.

## Methods

### Study design

We conducted a mixed methods study employing a concurrent triangulation design involving three arms. These arms include (i) facility survey of CHCs, (ii) in-depth interviews of CHC dentists, and (iii) household survey of randomly sampled households within one kilometre catchment area from CHCs. The availability of oral healthcare services at CHCs were studied through facility surveys (*n* = 12) and in-depth interviews with dentists (*n* = 12). The utilization of oral healthcare and corresponding factors were investigated through household survey. The data collection of the quantitative (facility survey, and household survey) and qualitative arms (in depth interviews) was conducted concurrently, triangulation of the findings was done at the interpretation phase [19]. 

### Study setting

**Fig. 1:**
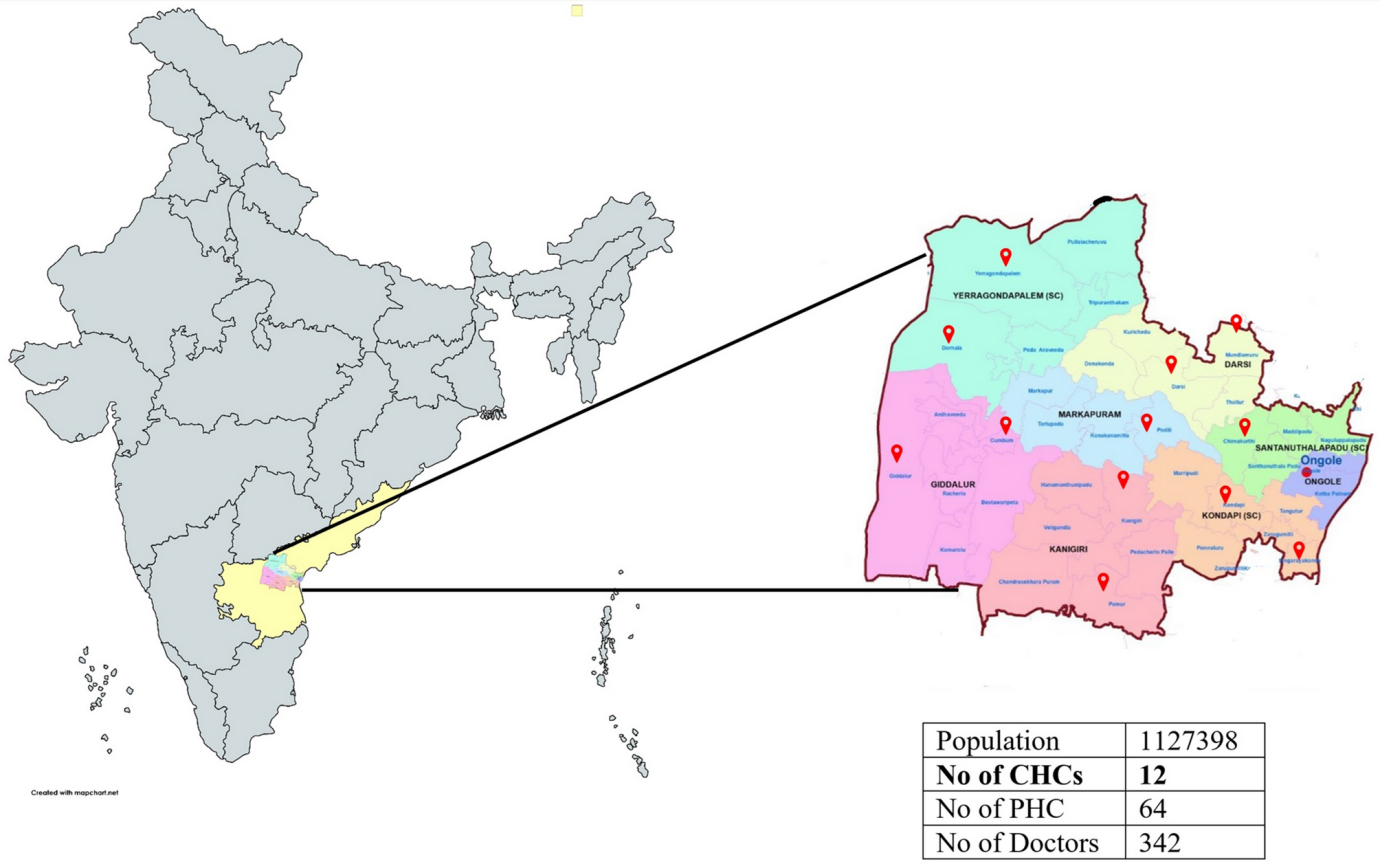
Geographical outline of the study sites. CHC = Community health centers, PHC = Primary health centers. Note: Population estimates based on population projections provided by the government agency. Doctors refer to doctors with a medical degree and working in public healthcare system

The study was conducted in Prakasam district of Andhra Pradesh, India. The district has over a million population, with its public healthcare system comprising of 64 PHCs, and 12 CHCs (see Fig. 1). This study included all the CHCs in the district. Study was conducted between January to June 2024.

### Sample size & sample selection

Considering the multi-arm nature of the study, multiple sample selection procedures were employed. For facility survey, all the CHCs in the district (*n* = 12) were included. For qualitative in-depth interviews, the dentists positioned at respective CHC were purposively sampled. Given that there was only one dentist per CHC, all the dentists working at CHC level (*n* = 12) were recruited. For the household survey, a sample size of 360 was estimated using Openepi (available at https://www.openepi.com/SampleSize/SSPropor.htm) based on a hypothesized proportion for oral healthcare utilization of 24% [11], a precision of 6%, a design effect of 1.5, a 95% confidence level, 0.05 ‘α’ and a non-response rate of 20%. The sample selection process followed a multi-stage cluster sampling approach. Given that distance impacts access and use [20, 21], an area within a one-kilometer radius of each CHC was considered as a cluster (primary sampling unit). From each cluster, 30 households (secondary sampling units) were sampled employing systematic random sampling, by selecting every 10th household from the CHC. Further one adult aged 18 years or above from each selected household was sampled employing KISH grid method [22]. 

Non-functional CHCs, CHCs where permission was not provided were exclusion criteria for facility survey. However, no CHC was excluded as all CHCs in the district (*n* = 12) were functional and provided the permission for facility survey. Qualitative interviews included consenting dentists working at the CHC level. The household survey included individuals aged 18 years and above & individuals who consented to participate in the study. The households which were locked, or wherein resident individuals refused/unable to provide consent were excluded from the study. When sampled household was excluded, the immediate next household was approached to substitute for the excluded household.

### Data collection

Data collection was structured by CHC clusters (CHC and its one-kilometre catchment area). Within each CHC cluster, data collection comprised of one facility survey, one in-depth interview, and survey of 30 households conducted over a period of 7 to 14 days.

The facility survey was conducted using a checklist developed based on the IPHS guidelines on provision of oral healthcare services at the CHC [7, 23]. The checklist captured data across indicator items on dimensions of human resources (dentists, dental assistants), infrastructure and materials (dental rooms, dental chairs, dental instruments), and service provision (patient foot falls, procedures) (see supplementary file 1).

Household survey was conducted using a questionnaire prepared by the research team based on existing literature [11, 12, 23, 24]. The survey captured information on (i) oral health symptoms, (ii) socio-demographic characteristics, (iii) perceptions about oral healthcare, (iv) awareness about oral healthcare services provided by CHC, and (v) utilization of oral healthcare services. Oral health symptoms were assessed by a trained dentist based on self-reported items to six common oral health symptoms (i.e., toothache, loose teeth, gingival bleeding while brushing, bad breath, stains, gingival sensitivity) [25]. Socio-demographic characteristics included age, gender, education, and socio-economic status. Socio-economic status was captured employing the modified Kuppuswamy scale [26]. Perceptions about oral healthcare included (a) perceived importance of oral health, (b) perception on cost of care, (c) attendance to oral health outreach programmes, and (d) perceived access to oral healthcare. The awareness about oral healthcare services provided by CHC was assessed using a six-item inventory with three-point Likert type items. Each item in the inventory had the responses “not aware” (coded as 0), “partially aware” (coded as 1), and “fully aware” (coded as 2), enabling computation of the composite score for awareness (see supplementary file 2). The six-items together had an acceptable level of internal consistency (coefficient α = 0.77). Utilization of Oral healthcare at CHC was captured using a single self-reported binary item, “*Ever visited CHC to seek oral healthcare in last one year*”. We ensured the content and face validity of the tools through an expert review process, in which independent external reviewers unaffiliated with the study team evaluated the tool’s items [27]. 

The in-depth interviews of CHC dentists were conducted by the first author who was an MPH candidate with a BDS qualification. The interviews were conducted face-to-face using semi-structured in-depth interview guide. The interviews were conducted in Telugu, the language spoken in study setting. The interview guide was developed based on author’s experience of interning with the district public healthcare system, literature review, and discussions with practicing dentists. The interview guide comprised of open-ended items capturing dentists’ views on availability, access, and utilization of oral healthcare services provided at CHC. Specifically, the interview guide captured the dentists’ views on provision of oral healthcare through CHC, financial, infrastructural and human resource constrains, community’s acceptance and engagement with oral healthcare services, and cultural/social factors influencing the uptake of services.

Prior to the interviews, the interviewer visited the CHC’s at least once, built rapport with the dentists, explained about the research being conducted, and obtained the consent to participate. The interviews were conducted at the dentists’ office and were audio recorded with consent of participants. All the CHC dentists consented to participate, with each interview lasting for approximately 30 min.

### Data analysis

The data from the facility survey were entered to Microsoft Excel^®^, and analysed descriptively. Frequencies were computed for the items on the nominal scale. For items on continuous scale (i.e., years of experience of dentist, average daily footfall), mean and standard deviation were computed.

The household data was cleaned and analysed using Statistical Package for Social Sciences (SPSS) version 27, and STATA version 13. Awareness level was computed based on composite index of six items assessing awareness about oral healthcare services provided through CHC. The scores ranged between 0 and 12. Individuals scoring “0” were categorized as “not aware about oral healthcare services”, and those scoring between 1 and 12 were categorised as “at least partially aware”. Socio-economic status was computed based on modified Kuppuswamy scale [26]. Univariate descriptive estimates were computed to report sample characteristics, awareness of oral healthcare services at CHC & their utilization. Multivariate analysis employing binary logistic regression was conducted to identify the factors associated with utilization of oral healthcare services provided by CHCs. Adjusted odds ratios (AOR) were computed, and the standard errors of AOR were adjusted for the cluster differences. The model fit was assessed using Hosmer lemeshow test for goodness of fit & discriminative ability of the model was computed by estimating the area under receiver operating characteristic (AUROC) curve.

The qualitative interviews were analysed employing a thematic analysis approach. The interviews were first transcribed and translated. Two authors independently read the interviews, familiarised and coded the interviews. An initial pool of codes was developed with a consensus between the coders, and they were used to further code the interviews. Semantic themes were developed from the codes, finally resulting in three overarching themes, and six sub-themes. Coding and theme development was conducted manually. Microsoft Excel^®^ was used to organize and manage the codes and facilitate theme development.

## Results

### Facility survey: availability of oral health care services at CHC

All the CHCs’ surveyed (*n* = 12) had an in-house dentist with a BDS qualification. The average years of experience of the dentists were 10.3 years. On average 13 patients visited dental OPD daily, and most common procedures provided at the CHCs were scaling and root planing, dental fillings and restorations, and oral health education (see Table 1).

**Table 1:** Findings of facility survey (*n* = 12)

Item Group	Indicator items	*N*/mean (± SD)
**Human Resources**	Dentist	12
	Educational Qualification (BDS)	12
	Dental Assistant	0
	Dental Hygienist	0
	Years of experience as a dentist	10.3 (**±** 7.9)
**Service Provision**	Daily Patient footfall at Dental OPD	13 (**±** 3.0)
	***Procedures Conducted***	
	Scaling and Root planing	12
	Disimpactions	8
	Emergency Cases^#^	0
	Root Canal treatments	0
	Extractions	7
	Dental Fillings and Restorations	12
	Oral Health Education	12
**Infrastructure**	Dental Room	12
	Dental Chair	12
	Dental X-Ray Machine	1
	Dental Instruments*	12
	Glass Ionomer Cement	12
	Calcium Hydroxide	12
	Povidone and iodine mouth washes	12
	Tooth Paste	12

BDS = Bachelor of Dental Surgery, OPD = Outpatient department, ^#^Include dental fractures/trauma cases *Dental instruments include mouth mirrors, periodontal probes, excavators

### Qualitative findings: factors influencing availability of oral healthcare services

**Fig. 2:**
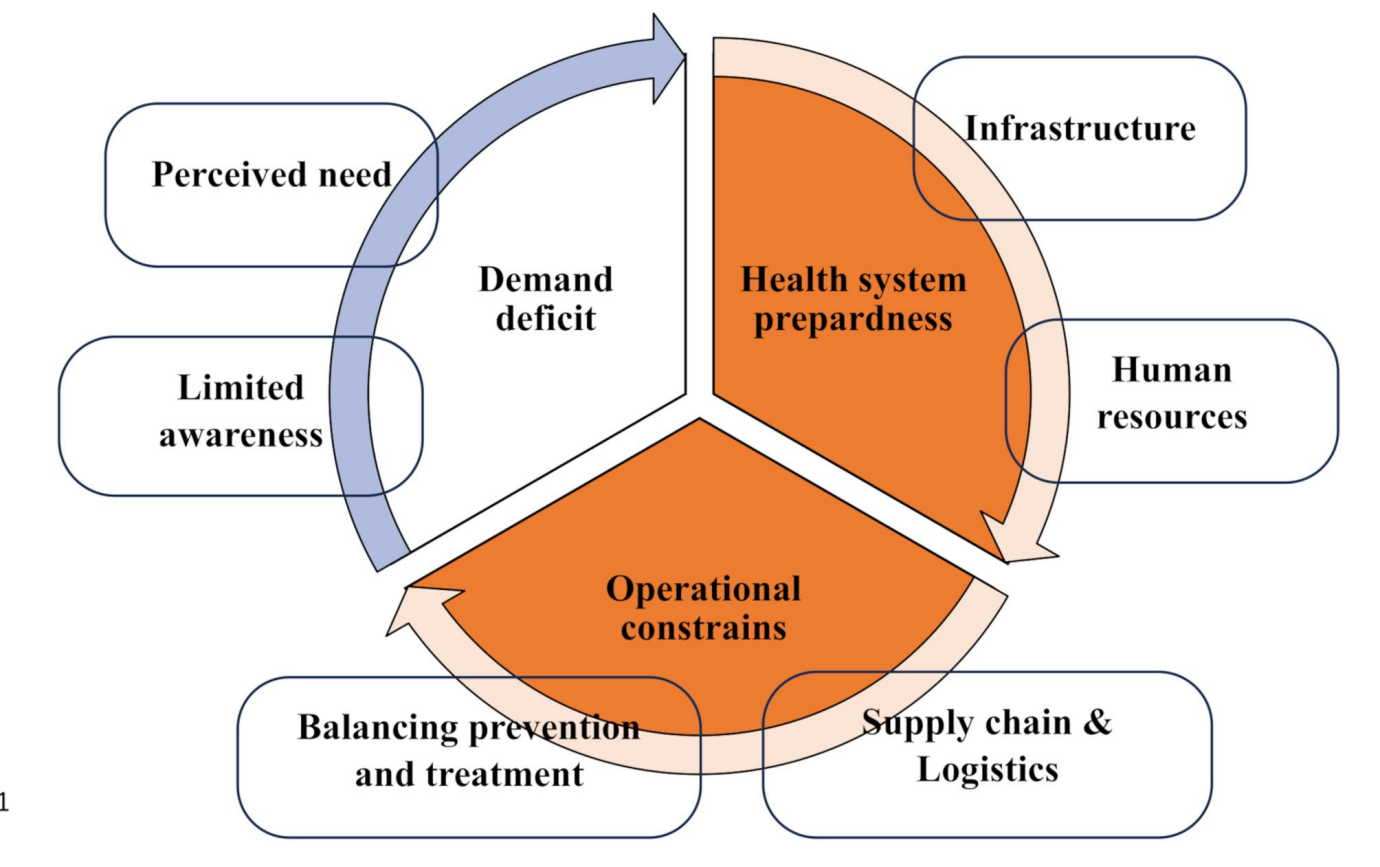
Overview of the themes developed from qualitative interviews

Three major themes explain the availability and utilization of oral healthcare services at CHCs, they include (i) demand deficit, (ii) health system preparedness, and (iii) operational constrains. The three themes and their constituent subcomponents reinforce each other thereby impacting the overall availability and utilization of oral healthcare services at CHCs (see Fig. 2).

### Theme 1: demand deficit

Despite the high prevalence of oral health symptoms, the demand for oral healthcare services remains low. Dentists interviewed reported that individuals often do not perceive oral health as important, leading them to neglect dental visits or attempt to soothe dental pain with home remedies or over-the-counter medications.

“*There is negligence towards oral health and people are not giving importance to oral health*”- Dentist 06.

“*People believe that keeping the substances like cloves near the buccal mucosa will take care of the problem*”- Dentist 03.

Moreover, the poor perception of oral health, combined with limited awareness about oral healthcare, has led to a demand deficit. The lack of knowledge about oral infections and the necessity of using oral healthcare services resulted in patients either not seeking treatment or being lost to follow-up.

“Most people do not have any kind of awareness and consequences of teeth infection…people neglect dental services. As the pain subsides, they don’t come back to CHC…after scaling and fillings, I will be giving an appointment to come on next day but they will not be coming back”-Dentist 11.

### Theme 2: health system preparedness

Dentists reported the non-availability of several essential oral healthcare services, particularly due to infrastructural limitations at the CHCs. The lack of key equipment and supplies, such as dental X-rays, local anaesthetics, and dental cements, prevented dentists from performing dental disimpactions, extractions, and root canal treatments. Furthermore, the unavailability of equipment and shortages of supplies led to the delivery of substandard care or the inability to provide care at all.

*“Due to lack of X-ray there was a delay of cases like extractions*,* and it has an impact on both doctor and patient”–* Dentist 01.

*“There is shortage of materials like cements and impression materials dental plaster*,* dental stone”-* Dentist 05.

In addition to infrastructural shortcomings, dentists reported an inadequate number of human resources, including dental hygienists, and dental technicians. Specifically, the near absence of these critical human resources severely limited the availability of oral healthcare services.

“*Absence of dental hygienists and dental assistants is one of the major drawbacks in providing services*”- Dentist 08.

### Theme 3: operational constrains

The access to oral healthcare at CHC was found to be limited by comparatively higher focus on oral health camps, and community awareness programmes. Dentists across the CHCs’ were of the opinion that while out-reach programmes were beneficial in terms of improving awareness, the limited resources and unilateral focus on preventive aspects of oral health limited the dentists’ ability to undertake treatment procedures at the CHC.

“*So the major caseload is observed when we go for camps and when we go to PHCs. People come there*,* we do screening*,* we ask them to come to CHC*,* and most of them don’t come. CHC is far from their place. Without manpower*,* without equipment*,* we cannot treat effectively at the PHC level*,* we are only able to do education programmes.*”- Dentist 04.

Further, we found pertinent supply chain issues, particularly with respect to procurement and supply logistics. Centralized procurement of dental supplies delinked from the need, and outreach activities not supported with necessary logistics limited oral healthcare delivery.

*“They have to ask doctors properly so that they know what is in shortage*,* what is necessary and send accordingly. For example*,* they sent the kidney trays multiple times*,* which is of no use. They could have utilized those funds for other materials like cement. Also*,* sometimes they send material like complete denture*,* without a technician how can we use those materials. They even sent reamers and files*,* when there is no X-ray machine so what can be done with them?”-*Dentist 12.

“*Sometimes we need to cover two out-reach camps per day. Places are far away*,* there is no transportation*,* no support. It is challenging*”- Dentist 01.

### Household survey: utilization of oral healthcare services provided by CHC

The household survey included 360 randomly sampled individuals. The study sample included approximately equal number of males and females, close to three-fourth of the survey sample are in the age groups 18–50 years, and a third of the sample had diploma, graduate or higher education qualification (see Table 2).

Oral health symptoms were highly prevalent among the study sample, and 79.8% (*n* = 285) reported having at least one of the six common oral health symptoms (tooth ache, loose teeth, gingival bleeding, bad breath, stains, gingival sensitivity). Further, gingival sensitivity was most commonly reported (50.3%) followed by stains (41.1%), tooth ache (40.8%), and bad breath (40.8%) (see supplementary Table 1).

Close to a fourth of respondents had no awareness about the CHCs and oral healthcare services provided by the CHC, with 70.8% reporting being ‘not aware’ that the dentist was available at CHC, and 91.1% not being aware that the CHC provides oral healthcare free of cost (see supplementary Table 2).

The utilization of oral healthcare was minimal with only 13.9% (*n* = 50) of the respondents of household survey reported visiting CHC to seek oral healthcare in the last one year.

**Table 2:** Outline of the characteristics of the sample in household survey (*n* = 360)

Study variables	*N*	%
**Age**		
18–35 years	131	36.4
36–50 years	137	38.1
51 years and above	92	25.6
**Gender**		
Female	179	49.7
Male	181	50.3
**Socio-economic status**		
Lower income households	49	13.6
Middle income households	110	30.6
Upper income households	201	55.8
**Education level**		
No formal schooling	151	41.9
Primary to high school	89	24.7
Diploma, graduate or higher education	120	33.3
**Oral health symptoms**		
No oral health symptoms	75	20.8
Any one oral health symptom	73	20.3
Multiple oral health symptoms	212	58.9
**Perception on importance of oral health**		
Some what important	166	46.1
Very important	194	53.9
**Perception on cost of oral care**		
Not affordable	271	75.3
Affordable	89	24.7
**Ever attended oral health outreach programmes**		
No	289	80.3
Yes	71	19.7
**Awareness about oral healthcare services at CHC**		
Not aware	87	24.2
At least partially aware	273	75.8
**Perceived access to dental care**		
Not accessible	306	85.0
Accessible	54	15.0
**Ever visited CHC to seek oral healthcare in last one year**		
No	310	86.1
Yes	50	13.9

**CHC** = Community Health Center

### Factors associated with utilization of oral healthcare services form CHC

Awareness about oral healthcare services at CHC (AOR = 10.35, 95% CI = 2.31–46.41), perceived access to oral healthcare (AOR = 4.89, 95% CI = 2.06–11.61), and reporting multiple oral health symptoms (AOR = 3.19, 95% CI = 1.03–9.90) were significantly associated with utilization of oral healthcare services from CHCs (see Table 3).

**Table 3:** Factors influencing use of oral healthcare services from CHC (*n* = 360)

Study variables	Ever visited CHC to seek oral healthcare in last one year		OR (95% CI)	AOR (95% CI)	*p*-value
	No(*n* = 310)	Yes(*n* = 50)			
**Age group**					
51 years and above (ref)	76	16			
18–35 years	117	14	0.57 (0.26–1.23)	1.16 (0.43–3.13)	0.769
36–50 years	117	20	0.81 (0.40–1.67)	1.38 (0.60–3.14)	0.447
**Gender**					
Male (ref)	156	25			
Female	154	25	1.01 (0.56–1.84)	1.21 (0.61–2.41)	0.588
**Socio-economic status**					
Lower income households (ref)	41	8			
Middle income households	97	13	0.69 (0.27–1.78)	0.65 (0.21–2.05)	0.465
Upper income households	172	29	0.86 (0.37–2.03)	0.75 (0.28–2.06)	0.581
**Education level**					
No formal schooling (ref)	130	21			
Primary to high school	74	15	1.26 (0.61–2.58)	1.46 (0.64–3.34)	0.374
Diploma, graduate or higher education	106	14	0.82 (0.40–1.69)	1.49 (0.51–4.35)	0.462
**Oral health symptoms**					
No oral health symptoms (ref)	70	5			
Any one oral health symptom	66	7	1.49 (0.45–4.91)	1.35 (0.36–5.11)	0.654
Multiple oral health symptoms	174	38	3.06 (1.16–8.09)	3.19 (1.03–9.90)	0.045
**Perception on importance of oral health**					
Somewhat important (ref)	140	26			
Very important	170	24	0.76 (0.42–1.38)	1.06 (0.52–2.15)	0.875
**Perception on cost of oral care**					
Affordable (ref)	234	37			
Not affordable	76	13	1.08 (0.55–2.14)	1.00 (0.45–2.25)	0.993
**Ever attended oral health outreach programmes**					
No (ref)	259	30			
Yes	51	20	3.39 (1.78–6.42)	1.94 (0.84–4.48)	0.120
**Awareness about oral healthcare services at CHC**					
Not aware (ref)	85	2			
At least partially aware	225	48	9.07 (2.16–38.13)	10.35 (2.31–46.41)	0.002
**Perceived access to dental care**					
Not accessible (ref)	276	30			
Accessible	34	20	5.41 (2.77–10.56)	4.89 (2.06–11.61)	< 0.001

Dependent variable: Ever visited CHC to seek oral healthcare in last one year: No (ref), Yes; ref = Reference; OR = Unadjusted odds ratio; AOR = Adjusted odds ratio; CI = Confidence interval; CHC = Community health centers; Hosmer Lemeshow test: 0.523, Nagelkerke R Square: 0.244

**Fig. 3:**
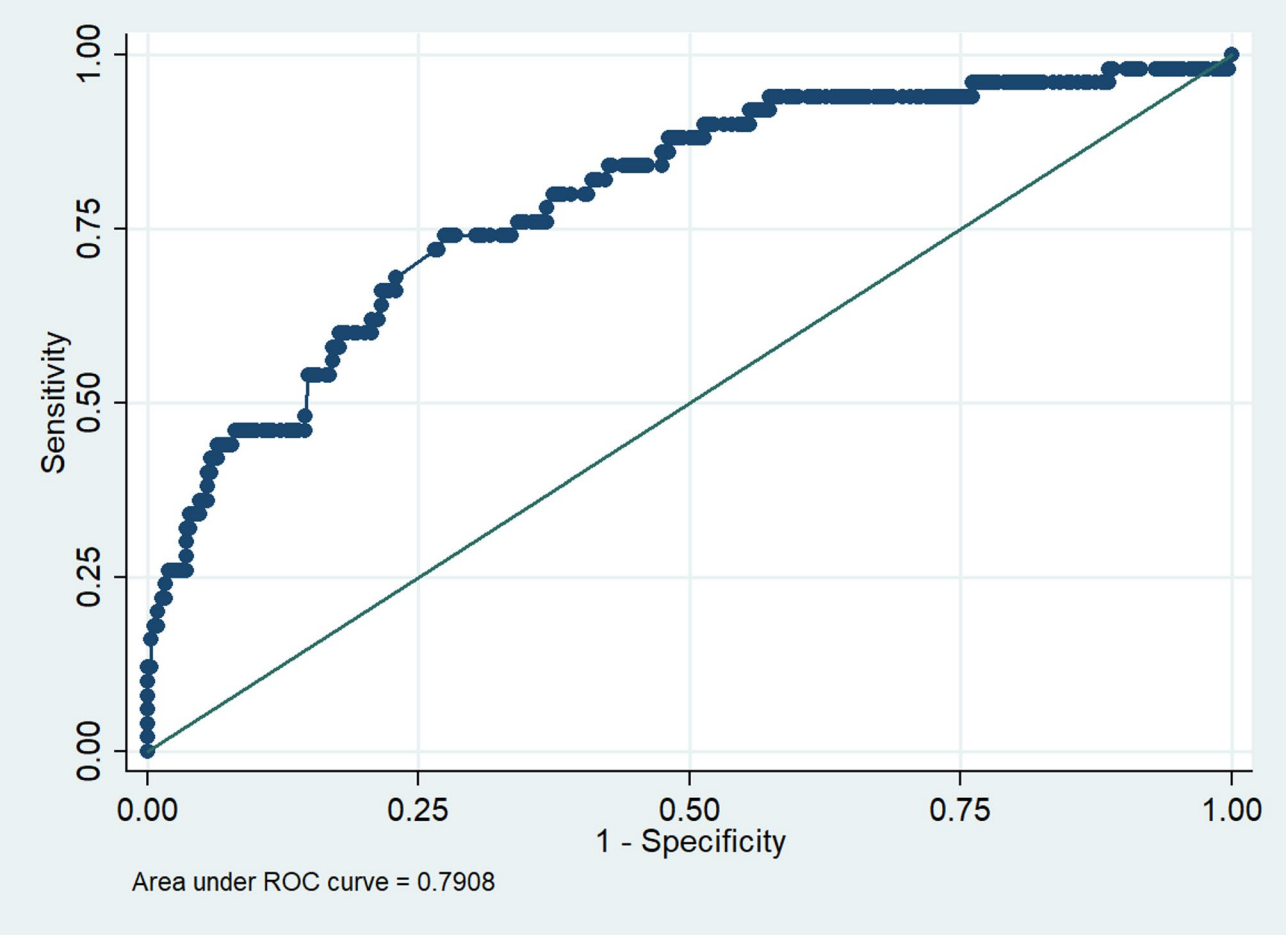
Discriminative ability of the regression model on utilization of oral healthcare at CHCs. ROC = receiver operating characteristic

The regression model had an acceptable discriminative ability with an AUROC of 0.791 (see Fig. 3). The model is highly specific with a specificity of 98.39%, positive predictive value of 70.59%, negative predictive value of 88.92%, with 88.06% of cases ascertained to be correctly classified by the model.

## Discussion

Our findings provide a comprehensive picture of the availability and utilization of oral healthcare services provided by CHCs at a district level in India. We found that all the CHCs had an in-house dentist and basic equipment indicating a better availability of dentists and oral healthcare infrastructure compared to what was previously reported at the PHC and CHC levels in other settings during the early years of NOHP implementation [28, 29]. The services provided however were limited to oral health education, scaling and root planing, and dental fillings and restorations concurring with previous studies [23] While the IPHS necessitate the provision of services such as root canal treatment, dental extractions, dental disimpactions, and treatment of emergency cases [7], our study and the previous studies found that there were shortcomings in provision of these services at CHC [23]. 

The limited availability of oral healthcare services was further explained in the in-depth interviews of dentists working at the CHCs. The dentists reported major challenges with respect to the preparedness of the healthcare system and operational constrains limiting their ability to deliver oral healthcare services. Non-availability of dental X-ray at the CHC level meant that critical procedures such as root canal treatments that rely on dental X-rays could not be adequately performed. Previous studies highlighted the same [12, 28]. Further low patient footfall, attributable to limited awareness (i.e., > 80% are unaware that CHC provides oral healthcare) [11, 14], and overburdening of dentists while managing competing priorities of dental OPD, day-procedures, oral health camps, and PHC outreach in settings with limited supportive manpower could have translated into inefficiencies in provision of oral healthcare. While literature among dentists practicing in public sector are limited, workload and health worker shortages were documented to hinder healthcare delivery in public sector healthcare facilities [30, 31]. Specifically, non-availability of dental hygienists and assistants remain a challenge. In Andhra Pradesh, institutions offering programmes to train dental hygienists are limited resulting in the positions lying vacant. While attempts have been made to recruit candidates with a BDS qualification in these positions [32], they are unsuccessful. Moreover, dentists reported of the existing supply chain issues wherein few essential consumables were practically unavailable, while some were supplied in excess. The procurement and distribution challenges in the public healthcare system were previously reported to hinder availability and access to essential medicines [33]. Our findings present a similar situation for oral healthcare supplies.

Our estimate of 13.9% utilizing oral healthcare from CHCs concur with the previous estimates of just around a quarter of the Indian population visiting a dentist, and limited utilization of oral healthcare in public sector [6, 11]. The average daily footfall of dental OPD was estimated to be less than the IPHS minimum requirement of 20 per day [7], and indicates a suboptimal utilization of oral healthcare services at CHC level considering their population coverage (approx. 120,000 per CHC) and high prevalence of oral health symptoms.

This low utilization could be further explained through Andersen’s healthcare utilization model [16] and the specific factors identified in our analysis. From the perspective of the ‘need factors’ presentation of multiple oral health symptoms increases likelihood of utilizing oral healthcare [34]. Dentists reporting substantial ‘demand deficit’ for their services, and close to half of the study sample not perceiving oral health as ‘very important’, reiterate that limited priority accorded to oral healthcare at an individual level is a major predisposing factor hindering its utilization [24, 35, 36]. Further, studies report that individuals visit a dentist as a last resort only when the symptoms worsen [24, 37, 38], substantiating our arguments.

While accessibility of oral healthcare services is an important enabler, close to 85% of our study sample reported oral healthcare as ‘not accessible’. Previous research reported the components such as cost of care, distance to travel, and working hours of OPD influenced access [37, 39, 40]. Unlike earlier studies [37, 39, 40], the economic variables (i.e., socio-economic status of household and perceived cost of care) were not significantly associated in our study. The focus of the study (i.e., oral healthcare delivered through CHCs), and composition of our study sample (majorly middle- and upper-income households) may have resulted in the difference. Receiving care at CHCs is free of cost, and middle- and upper-income groups are known to prefer private healthcare providers [41], resulting in the muted effect of the economic variables. Also, while at least being partially aware about oral healthcare services provided by CHC significantly enabled their utilization [42–44], overall awareness levels remain poor, with < 10% being aware that CHCs provide oral healthcare free of cost, reflecting a substantial untapped demand.

Improving oral health is crucial for advancing the Universal Health Coverage agenda [45]. In India, disparities in dental care access stem from limited public provision, infrastructural challenges, low utilization of services, and widespread lack of awareness [46]. Targeted awareness initiatives such as expanded outreach programs, engagement of community health workers, and innovative advertising campaigns modelled after successful maternal and child health interventions could be piloted to improve demand for oral healthcare services [47, 48]. Addressing systemic challenges through strategic investments in providing essential oral healthcare infrastructure, improved supply chains (through distribution hubs, public-private partnerships and involvement of dentists in procurement process), recruitment of dental staff and task-shifting approaches (such as training nurses to take up the role of dental assistants) could improve oral healthcare delivery through CHCs.

### Limitations

The study is limited to oral healthcare provided through rural CHCs in one district in a south Indian state. Dentist availability in India’s public healthcare system is varied, with several states facing a shortfall. Our findings may not be valid in such contexts. Our household survey sample comprised majorly of middle income and upper income classes, which could have resulted in a lower rate of oral healthcare use at CHCs. Owing to logistical constrains we did not conduct clinical examination or in-depth interviews of household survey participants. We also did not explore the participant’s preference on using oral healthcare services from public healthcare facilities. Our study is limited by its cross-sectional nature, and potential recall/self-reported bias. Nevertheless, our study provides formative evidence on oral healthcare availability and utilization at CHCs, and serves as basis for further studies to strengthen the public provision of oral healthcare. Future research is required to further examine the patients’ perspective to utilizing the oral health care services. Given that public provision of oral healthcare services in India is still at a nascent stage, longitudinal studies are required to further investigate these trends.

## Electronic supplementary material

Below is the link to the electronic supplementary material.

Supplementary Material 1

Supplementary Material 2

Supplementary Material 3

Supplementary Material 4

## Data Availability

The datasets used and/or analysed during the current study are available from the corresponding author on reasonable request.
