# Supplementary material for: Availability and utilization of oral healthcare services at rural community health centers in South India: a mixed methods study
Source: BMC Oral Health. 2025 Jul 1;25:977. doi: 10.1186/s12903-025-06327-1 (PMC12220351; doi:10.1186/s12903-025-06327-1)
Supplement: Supplementary file 1 — Supplementary Material 1 [file 12903_2025_6327_MOESM1_ESM.docx]

**Checklist for Facility Survey**

1. Name of the CHC:
2. **Human resources**

| Dentist | Dental Assistant | Dental Hygienist |
| --- | --- | --- |
| 1. Educational Qualification: | 1. Educational Qualification: | 1. Educational Qualification: |
| 2. Specialisation if any : | 2. Specialisation if any : | 2. Specialisation if any : |
| 3. Years of Experience : | 3. Years of Experience : | 3. Years of Experience : |
| 4. Years since joining CHC: | 4. Years since joining CHC: | 4. Years since joining CHC: |

1. **Services Provided**

| Types of services | Response |
| --- | --- |
| 1)      Dental OP (Minimum 20 pts per day) | Yes/No |
| 2)      Patient footfall at Dental OPD as on the day of survey |  |
| 3)      Dental procedures (8-10 per day) | Yes/No |
| 4)      Emergency cases* | Yes/No |
| 5)      Scaling and Root planning | Yes/No |
| 6)      Disimpactions | Yes/No |
| 7)      Root canal treatments | Yes/No |
| 8)      Extractions | Yes/No |
| 9)      Fillings or restorations | Yes/No |
| 10)  Oral health education | Yes/No |

*Trauma cases

1. **Infrastructure and materials**

| **Infrastructure and material** | **Not available** | **Available** |
| --- | --- | --- |
| 1.Dental Room |  |  |
| 2.Dental Chair |  |  |
| 3.Dental X-ray machine |  |  |
| 4.Mouth Mirrors |  |  |
| 5.Peridontal Probes |  |  |
| 6.Excavators |  |  |
| 7.Glass Inomer Cement |  |  |
| 8.Calcium hydroxide |  |  |
| 9. Povidone and iodine mouth washes |  |  |
| 10.Tooth paste |  |  |
