# Supplementary material for: Availability and utilization of oral healthcare services at rural community health centers in South India: a mixed methods study
Source: BMC Oral Health. 2025 Jul 1;25:977. doi: 10.1186/s12903-025-06327-1 (PMC12220351; doi:10.1186/s12903-025-06327-1)
Supplement: Supplementary file 2 — Supplementary Material 2 [file 12903_2025_6327_MOESM2_ESM.docx]

**Household Survey Questionnaire**

**Oral health status**

- 1. Do you currently have any oral health issues?

Yes

No

- 1. Have you experienced toothache in the past month?

Yes

No

- 1. Do you currently have any loose teeth?

Yes

No

- 1. Have you noticed gingival bleeding when brushing your teeth?

Yes

No

- 1. Do you frequently experience bad breath?

Yes

No

- 1. Have you observed stains on your teeth that concern you?

Yes

No

- 1. Do you often experience gingival sensitivity (sensitivity of the gums)?

Yes

No

**Socio-demographic characteristics**

- 1. Age of the participant ____________________________________
  2. Gender: Male/Female
  3. Education level of the individual

No formal schooling

Primary School

Secondary Schooling

High School

Diploma

Graduate

Post graduate/University/Higher

**Socioeconomic status of the household**

- 1. Highest Educational level of the head of the family:

Profession or honours

Graduate

Intermediate or diploma

High school certificate

Middle school certificate

Primary school certificate

Illiterate

- 1. Occupation of the head

Legislators, senior officials, and managers

Professionals

Technicians and associate professionals

Clerks

Skilled workers and shop and market sales workers

Skilled agricultural and fishery workers

Craft and related trade workers

Plant and machine operators and assemblers

Elementary occupation

Unemployed

- 1. Monthly family income in Indian Rupees:

≥20,482

10,241–20,481

7681–10,240

5120–7680

3072–5119

1034–3071

≤1033

- 1. Socioeconomic class based on the items of modified Kuppuswamy scale (items 4, 5, 6)

Upper income households

Upper middle-income households

Lower middle-income households

Upper lower income households

Lower income households

**Perceptions about oral healthcare**

- 1. How do you perceive the importance of oral health?

Somewhat important (0), Very important (1)

- 1. What is your view on cost of accessing the oral health care?

Affordable (1), Not affordable (0)

- 1. Have you ever attended dental camps or oral health outreach programmes?

Yes (1), No (0)

- 1. How do you perceive the access to dental care in your community?

Accessible (1), Not accessible (0)

**Awareness about oral healthcare services provided at CHC**

- 1. Aware about CHC in the vicinity

Not aware (0), Partially aware(1), Fully aware (2)

- 1. Aware about the presence of dentist at CHC

Not aware (0), Partially aware(1), Fully aware (2)

- 1. Aware that CHC provides oral healthcare services

Not aware (0), Partially aware(1), Fully aware (2)

- 1. Aware of health programmes delivered through CHC

Not aware (0), Partially aware(1), Fully aware (2)

- 1. Aware of any oral health education programmes conducted in your community

Not aware (0), Partially aware(1), Fully aware (2)

- 1. Aware that the CHC provide oral healthcare free of cost

Not aware (0), Partially aware(1), Fully aware (2)

**Oral healthcare utilization:**

- 1. Have you ever visited CHC to seek oral healthcare in last one year?

Yes (1) , No (0)
