# Supplementary material for: Availability and utilization of oral healthcare services at rural community health centers in South India: a mixed methods study
Source: BMC Oral Health. 2025 Jul 1;25:977. doi: 10.1186/s12903-025-06327-1 (PMC12220351; doi:10.1186/s12903-025-06327-1)
Supplement: Supplementary file 3 — Supplementary Material 3 [file 12903_2025_6327_MOESM3_ESM.docx]

**Supplementary Tables**

Supplementary Table 1

| **Oral health symptoms** | **n** | **%** |
| --- | --- | --- |
| Tooth ache | 147 | 40.8 |
| Loose teeth | 113 | 31.4 |
| Gingival bleeding | 134 | 37.2 |
| Bad breath | 147 | 40.8 |
| Stains | 148 | 41.1 |
| Gingival sensitivity | 181 | 50.3 |

Supplementary Table 2

| **Awareness Items** | **Not aware**  **(0)** | **Partially aware (1)** | **Aware (2)** |
| --- | --- | --- | --- |
|  | **%** | **%** | **%** |
| Aware about CHC in the vicinity | 26.4 | 21.7 | 51.9 |
| Aware about the presence of dentist at CHC | 70.8 | 15.3 | 13.9 |
| Aware that CHC provides oral healthcare services | 82.2 | 11.7 | 6.1 |
| Aware of health programmes delivered through CHC | 85.3 | 11.9 | 2.8 |
| Aware of oral health education programmes | 87.8 | 10.3 | 1.9 |
| Aware that the CHC provide oral healthcare free of cost | 91.1 | 7.8 | 1.1 |
