# Supplementary material for: Availability and utilization of oral healthcare services at rural community health centers in South India: a mixed methods study
Source: BMC Oral Health. 2025 Jul 1;25:977. doi: 10.1186/s12903-025-06327-1 (PMC12220351; doi:10.1186/s12903-025-06327-1)
Supplement: Supplementary file 4 — Supplementary Material 4 [file 12903_2025_6327_MOESM4_ESM.pdf]

केरल केंद्रीय विश्वविद्यालय
CENTRAL UNIVERSITY OF KERALA

(संसद के अधिनियम, वर्ष 2009 द्वारा स्थापित / Established under the Act of Parliament in 2009)

Institutional Human Ethics Committee

IHEC/CUK/2023/69

10th January 2024

Panditi Madhuri

MPH (III Semester), Department of Public Health and Community Medicine

The Institutional Human Ethics Committee reviewed and discussed your application for the study entitled, "Availability and Utilisation of dental services at Community Health Centers in Prakasam district of Andhra Pradesh" on 18th and 19th December 2023

The following members of the ethics committee were present in the meeting.

Sl. No	Member Name	Highest Degree	Gender	Scientific/Non-Scientific	Affiliation with the Institution
1	Dr. Jayakrishnan Thavody	MD	Male	Chair Person (Clinician/Ethicist)	No
2	Adv. Shreekath K	LLB	Male	Legal Expert	No
3	Dr. Thejaswini Venkatesh	PhD	Female	Biomedical expert	Yes
4	Sri. Rajeevan CP	MA	Male	Philosopher	No
5	Mr. Shylendran MV	MA	Male	Lay Person	No
6	Dr Nagalingam M	PhD	Male	Social Scientist	Yes
7	Prof.RajendraPilankatta	PhD	Male	Biomedical Expert	Yes
8	Dr. Prakash Babu Kodali	PhD	Male	Member Secretary (Public Health Expert)	Yes

With reference to the minutes of the Institutional Human Ethics Committee (IHEC) meeting conducted on 18th and 19th December, the committee approved your study in the present form. You are requested to inform the IHEC upon the completion of the study/any modifications made to the approved research. The IHEC is organized and operated according to the requirements of Indian Council of Medical Research (ICMR) and is provisionally registered with the National Ethics Committee Registry for Biomedical and Health Research (NECRBHR), Department of Health Research (DHR), Government of India.

Member Secretary
Institutional Human Ethics Committee
Central University of Kerala
